# Long-Term Efficacy of Ultrasound-Guided Percutaneous Laser Ablation for Low-Risk Papillary Thyroid Microcarcinoma: A 5-Year Follow-Up Study

**DOI:** 10.1155/2021/6616826

**Published:** 2021-07-23

**Authors:** Kai Peng, Ping Zhou, Wengang Liu

**Affiliations:** Department of Ultrasound, The Third Xiangya Hospital, Central South University, Changsha, Hunan, China 410013

## Abstract

**Objective:**

To evaluate the long-term efficacy and safety of ultrasound-guided percutaneous laser ablation (PLA) for the treatment of low-risk papillary thyroid microcarcinoma (PTMC).

**Methods:**

From June 2012 to May 2015, 105 patients with solitary, pathologically confirmed PTMC lesions were treated with ultrasound-guided PLA. Nodule location, nodule volume, thyroid function, and clinical symptoms were evaluated before ablation. Contrast-enhanced ultrasound (CEUS) was performed 1 h after treatment to evaluate whether the ablation was complete. Ultrasound examination was performed at 1, 3, 6, and 12 months after ablation and every 6 months thereafter to determine the size of the ablation area and search for recurrence in the thyroid parenchyma and lymph node metastasis. Thyroid function was examined before and 1 month after ablation. Fine needle aspiration biopsy was performed for any suspicious metastatic lymph nodes and recurrent lesions in the thyroid.

**Results:**

All 105 lesions were completely inactivated after one ablation, making the success rate for single ablation 100%. The average ablation time was 2.78 ± 1.05 min, and the average ablation energy was 505 ± 185 J. All patients could tolerate and complete the ablation. No serious complications occurred during the treatment; only minor side effects such as pain and local discomfort were reported. The volume reduction rates were −781.14 ± 653.29% at 1 h posttreatment and −268.65 ± 179.57%, −98.39 ± 76.58%, 36.78 ± 30.32%, 75.55 ± 21.81%, 96.79 ± 10.57%, and 100% at 1, 3, 6, 12, 18, and 24 months after ablation, respectively. This rate remained 100% at the later follow-up times. Overall, 28 (26.67%), 74 (70.48%), 96 (91.43%), and 103 (100%) were completely absorbed by 6, 12, 18, and 24 months after PLA. One patient developed another lesion 12 months after ablation, and two patients had central cervical lymph node metastasis 24 months after ablation.

**Conclusion:**

PLA is a safe and effective alternative clinical treatment for low-risk PTMC.

## 1. Introduction

In recent years, due to the wide application of high-resolution ultrasound and ultrasound-guided fine needle aspiration biopsy (FNAB), the detection rate of papillary thyroid microcarcinoma (PTMC) has increased [1, 2]. Now, more than 50% of new cases of thyroid cancer are PTMC [3]. At present, surgery is still the most commonly used treatment method in clinical practice, but serious postoperative complications, such as parathyroid injury and recurrent laryngeal nerve injury, [4–7], remain a major challenge. In addition, some cases are not suitable and some patients are unwilling to accept surgery for reasons such as physical contraindications for surgery, aesthetic requirements for some occupations, and personal wishes. PTMC is small in size, slow to develop, and low in biological malignancy. As such, it rarely progresses to thyroid cancer with clinical significance, and some patients can live a lifetime with PTMC without symptoms. Even the presence of clinical symptoms or regional lymph node metastasis has been shown to have little impact on the survival rate among PTMC patients [8, 9]. Therefore, active surveillance rather than treatment is recommended for asymptomatic and nonmetastatic PTMC cases. However, active surveillance has some drawbacks: first, although PTMC has a good prognosis, some cases will not maintain a subclinical status without progression, and some cases will have highly invasive histological types, resulting in regional or distant metastasis in the early stage [10, 11]. Second, patients with suspicious nodules on ultrasonography- or biopsy-proven PTMC tend to experience anxiety that can create an obstacle for successful long-term surveillance and may decrease the patient's quality of life.

As one of the most minimally invasive techniques for the removal of in situ tumors, ultrasound-guided percutaneous laser ablation (PLA) has been clinically applied for the treatment of various benign and malignant tumors [12–15]. Similar to ultrasound-guided radiofrequency ablation (RFA) and microwave ablation (MWA), PLA can generate a high temperature locally through energy, which leads to tissue thermal denaturation and coagulative necrosis. However, unlike that in RFA and WMA, the range of energy effect per unit time for PLA is small, which makes this approach safer with more precise energy concentration [16, 17]. The emission form of PLA energy is forward, which causes little damage to the lateral tissues of the laser fiber. Therefore, compared with chemical interventional therapy and other thermal ablation therapies, PLA can offer better safety and efficacy for the treatment of small lesions. Traditionally, PLA has been mainly used in the treatment of benign thyroid nodules [18–20], with long-term follow-up showing that abnormal thyroid function was significantly improved, the volume of nodules was significantly reduced, and the therapeutic effect was significant [21, 22]. However, research regarding the use of ultrasound-guided PLA for the treatment of thyroid cancer is still in the initial stage. The purpose of this study was to study the efficacy and safety of ultrasound-guided PLA for the treatment of PTMC, in order to provide a basis for the clinical treatment of PTMC with PLA.

## 2. Materials and Methods

### 2.1. Patients

Patients with PTMC who were treated by ultrasound-guided PLA in our department from June 2012 to May 2015 were screened for inclusion in this study. The inclusion criteria were as follows: (1) ultrasound showed a single nodule with a maximum diameter of ≤1.0 cm that was not close to the capsule (distance ≥ 2 mm) as well as no sign of cervical lymph node or distant metastasis, (2) FNAB or core needle biopsy (CNB) confirmed the diagnosis of PTC, (3) the patient either could not tolerate or chose to reject surgical treatment, and (4) the patient required minimally invasive intervention due to mental concerns affecting their normal life and refusal of active surveillance. Patients who met any of the following exclusion criteria were excluded from this study: (1) observation of a suspicious metastatic cervical lymph node was observed, (2) gross calcification appearing in the lesion, (3) abnormal contralateral vocal cord function, (4) presence of a severe coagulation disorder, or (5) presence of severe cardiopulmonary disease.

The study was approved by the ethics committee of our hospital. After consultation regarding the PLA method, process, postablation considerations, and the possibility of complications preablation, all patients provided written informed consent for treatment by PLA.

### 2.2. Preablation Assessment

All patients received conventional ultrasound, CEUS, and thyroid function examinations before ablation. The size, volume, ultrasound characteristics, and blood flow condition of the nodules were recorded by conventional ultrasound. Conventional ultrasound was also used to search for suspicious metastatic lymph nodes in the neck. CEUS was mainly used to evaluate the microvascular perfusion of the nodules. Nodule volume was calculated as follows: *V* = *π* · *a* · *b* · *c*/6, where *V* is the volume, *a* is the largest diameter of the nodule, and *b* and *c* are the other two perpendicular diameters of the nodule, respectively.

### 2.3. PLA Procedure

PLA was performed with an Esaote EchoLaser integrated ultrasonic diagnosis and laser ablation system (EcholaserX4, Elesta, Florence, Italy). The MyLab Twice instrument (Esaote, Elesta), with a LA332 high-frequency linear array probe, was used at a frequency of 3.0–11.0 MHz. The laser therapeutic apparatus was an EchoLaser X4 laser treatment system (EcholaserX4, Elesta). The Nd : YAG (neodymium-doped yttrium aluminum garnet) laser wavelength was 1064 ± 10 nm. Each fiber was 15 cm in length and 300 *μ*m diameter, with a maximum output power of 7 W ± 20%.

For application of PLA, patients were supine position with their neck fully exposed. Local anesthesia with 2% lidocaine was injected after disinfection of the area with a towel. The decision to apply isolation fluid to isolate important tissues and organs was made according to the location of the nodule. The isolation solution was a mixture of 2% lidocaine and normal saline (1 : 8 dilution). Under ultrasound guidance, the 21 g guide needle was injected into the center of the nodule to be treated, and then, the optical fiber was inserted into the same position from the needle core before the guide needle was pulled back 5 mm to leave the tip of the optical fiber in the original position and in direct contact with the tissue. The laser source parameters were set at a wavelength of 1064 nm and power of 3 W, and the laser transmitter was turned on to start the treatment. Under ultrasound real-time monitoring of the whole treatment process, with the release of the laser light source energy, an irregular strong echo gasification area would appear at the tip of the optical fiber and continue to expand. The treatment end point was when the ablation area completely covered and then exceeded the nodule area. After the treatment, the laser power supply was turned off, and the treatment time, total energy released by the laser, and any complications/side effects were recorded.

### 2.4. Evaluation of Ablation Success

CEUS was performed 1 hour after ablation to evaluate the curative effect of PLA, which was based on whether the nodule was completely ablated. The dynamic double contrast-imaging model was adopted with contrast-tuned imaging. The mechanical index was 0.03. The contrast agent was a second-generation SonoVue (Bracco, Milan, Italy), i.e., sulfur hexafluoride microbubbles enclosed by phospholipids. Twenty-five milligrams of SonoVue was diluted in 5 mL saline (0.9% Na Cl), and after vibration for 30 s, a microbubble suspension was created. The suspension was injected as a bolus through the antecubital vein with a 20 G trocar, and each 2.4 mL injection was then flushed with 5 mL saline. The dynamic perfusion of the lesion was continuously observed for 90 s, and the images were recorded. The presence of residual tissue was determined by the enhanced signal within the ablated lesion, through comparison with the preablation image to evaluate whether the ablation area covered and exceeded the original lesion. If the ablation area did not exceed the lesion area, additional ablation was required.

### 2.5. Evaluation of Long-Term Treatment Efficacy

The patients were followed up at 1, 3, 6, and 12 months after PLA and then every 6 months thereafter. Follow-up examinations included conventional ultrasonography, CEUS, and thyroid function measurement. Conventional ultrasonography and CEUS were performed to evaluate the size of the ablation area and search for recurrence in the thyroid parenchyma and lymph node metastasis. FNAB was performed for all lymph nodes suspected to be metastatic as well as suspicious lesions in the thyroid parenchyma. The volume reduction rate (VRR) was calculated as VRR = [(preablation volume–volume at follow‐up)/preablation volume] × 100%. Thyroid function was tested before and 1 month after ablation. The occurrence of related complications as well as the corresponding treatment and recovery details was recorded.

### 2.6. Statistical Analysis

Statistical analyses were performed using SPSS for Windows software (version 23.0, SPSS, Chicago, IL, USA). The measurement data are presented as mean ± standard deviation, and count data are presented as percentage and frequency. The paired *t-*test was used to identify changes in the nodule volume at each follow-up examination relative to the pretreatment volume. Significance was defined by *P* < 0.05.

## 3. Results

### 3.1. Clinical Characteristics of PTMC Patients

**Figure 1:**
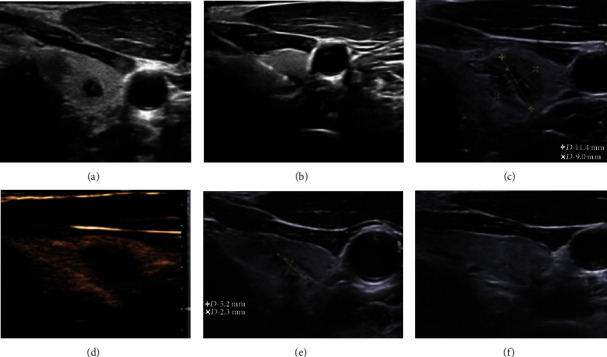
Representative ultrasound images of a PTMC nodule in the left thyroid lobe of a 32-year-old woman before and after ultrasound-guided PLA. (a) Before PLA, the hypoechoic nodule measured 5.1 mm × 3.9 mm × 4.1 mm in size and 42.7 mm^3^ in volume. (b) The laser fiber was inserted into the nodule through the guide needle, and the ablation was started at an output power of 3 W. Immediately, the nodule was completely covered by a patchy hyperecho gasification area. (c) One hour after PLA, the ablation area measured 11.4 mm × 9.0 mm × 10.5 mm, covering an area larger than the original area of the nodule. (d) One hour after PLA, no contrast agent filled the ablated region on CEUS, indicating complete ablation. (e) At 3 months after PLA, the ablation area was decreased to 5.2 mm × 2.3 mm × 3.1 mm. (f) At 6 months after PLA, the ablation area had been completely absorbed. No recurrence or lymph node metastasis was found during 5 years of follow-up.

A total of 105 patients with solitary PTMC lesion treated by ultrasound-guided PLA in our department from June 2012 to May 2015 were enrolled in this study. These patients included 74 women and 31 men with an average age of 44.1 ± 12.2 years (range, 23–68 years). Of the 105 lesions, 61 were located on the right side and 44 were located on the left side. The average maximum lesion diameter was 6.34 ± 2.62 mm (range, 4.5–9.8 mm), and the average lesion volume was 99.42 ± 84.01 mm^3^ (range, 20.28–416.56 mm^3^). The average ablation time was 2.78 ± 1.05 min, and the average total ablation energy was 505 ± 185 J (Table 1, Figure 1).

### 3.2. Postablation Imaging Appearance of Thyroid Nodules

All nodules were completely covered by an area of patchy hyperechoic vaporization immediately after PLA on two-dimensional grayscale images. One hour after ablation, the nodules showed heterogeneous mixed acoustic areas, and “equal sign” parallel patches appeared as hyperechoic bands without posterior acoustic shadowing in the center. CDFI showed that the color blood flow signal had disappeared in the ablation area. CEUS showed no contrast agent perfusion in the ablation area, and the contrast agent-filled defect in the ablation area was larger than the original nodule contrast enhancement area.

### 3.3. Complications and Side Effects

All patients could tolerate and complete the ablation, and no serious complications, such as trachea, esophagus, blood vessel, or laryngeal nerve injury, occurred after treatment. Sixty-eight patients (64.76%) complained of varying degrees of neck pain during the operation, but all reported that the pain was tolerable. Once the ablation was stopped, the symptoms became less intense or disappeared. Twenty-seven patients (25.71%) experienced neck pain and radiation pain in the head and face within 24 h after PLA, and these side effects were relieved at 1 week after treatment.

All patients had normal thyroid function before ablation. When thyroid function was reexamined 1 month after PLA, one patient had a slightly decreased thyroid-stimulating hormone (TSH) level, which returned to normal at the 3-month follow-up. One other patient had an elevated thyroglobulin antibody level, which recovered to normal by the 6-month follow-up. No drug treatment was given for abnormal thyroid function during this period.

### 3.4. Efficacy of PLA for Reducing PTMC Nodule Volume

**Figure 2:**
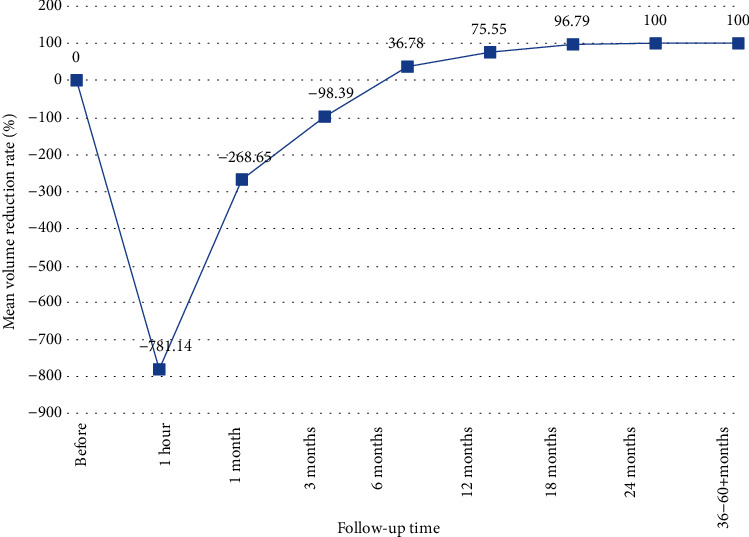
Mean ablation zone volumes for PTMC cases before and at each follow-up time after treatment of nodules with ultrasound-guided PLA.

The average volume of the 105 nodules before ablation was 99.42 ± 84.01 mm^3^, and due to the expansion of lesions during the ablation, the average nodule volume at 1 h after PLA was increased to 1067.23 ± 868.76 mm^3^. However, the volume of the nodules continued to decrease at later follow-up times until a VRR of 100% and complete absorption were observed for 103 lesions at 24 months posttreatment and thereafter (Table 2). Two cases did not complete the 24-month and later follow-ups because these two patients were found to have cervical lymph node metastasis at 24 months after PLA, and both received open surgical treatment (Figure 2).

### 3.5. PTMC Recurrence and Metastasis after Ultrasound-Guided PLA Treatment

Throughout the 5-year follow-up period, two patients were found to have central cervical lymph node metastasis at 24 months after PLA, and both received open surgical treatment. In another patient, a hypoechoic nodule measuring 4.6 × 3.1 × 4.0 mm around the area of the original nodule was observed at 12 months after ablation. The new suspicious nodule was confirmed to be PTMC by FNAB, and no obvious lymph node metastasis was found by imaging examination. The patient requested a second laser ablation. Over 12 months of follow-up, both lesions were fully absorbed, and no recurrence or lymph node metastasis occurred during the subsequent 3 years of follow-up. No obvious recurrent lesions or lymph node metastasis were observed in any of the other patients, and thus, the recurrence-free survival rate after ultrasound-guided PLA for PTMC in this study was 97.1%.

## 4. Discussion

Since Pacella et al. [23] introduced the use of PLA in the treatment of thyroid nodules, subsequent studies have confirmed the safety and efficacy of this treatment method [24–26]. Originally, PLA was used primarily in the treatment of benign thyroid nodules and only rarely used in the treatment of thyroid cancer. Over the past few years, several investigators have applied PLA as an alternative therapy to surgery in the treatment of PTMC, either as the only therapy applied or in combination with other treatments. Papini et al. [27] applied PLA treatment in one case of PTMC and achieved good results. In their case, FNAB and CNB performed 1 and 12 months after PLA revealed necrotic material and inflammatory cells with no viable neoplastic cells, and no lymph node or distant metastasis was found during 24 months of follow-up after PLA. Valcavi et al. [28] applied PLA before total thyroidectomy in three PTMC cases, and histological analysis proved the absence of residual viable tumor cells after PLA, supporting the effectiveness of PLA treatment. Good effectiveness of PLA for PTMC was reported by both Ji et al. [29], who treated 37 cases of PTMC with PLA, and Zhou et al. [30], who treated 30 PTMC patients with PLA. However, with only a few studies reported, the application of PLA in the treatment of PTMC has still been very limited. In addition, these previous studies either had small sample sizes or no long-term follow-up.

In the present study, 105 patients with PTMC were treated by PLA and followed up for at least 60 months. Compared with previous studies, our study had a greater sample size and longer follow-up. At 1 h after PLA, the mean maximum diameter and volume of nodules were significantly larger than those pretreatment, which indicated effective ablation of the lesions by PLA. During the follow-up, conventional ultrasound confirmed the disappearance of blood perfusion within the ablation area, and CEUS showed no contrast agent perfusion in the ablation area, further confirming that PLA achieved effective ablation of the lesions. During the first 2 years of follow-up, the ablation area continued to shrink, and by the 24-month follow-up, 103 ablation lesions had completely disappeared. The absorption rate observed in the present study is higher than that reported by previous studies, and this outcome is likely due to the longer follow-up time in the present study.

The main complications of PLA treatment for thyroid nodules include pain, dysphoria, bleeding, cough, vagal reaction, infection, and self-limited hyperthyroidism, but the rate of complications is very low [12, 31–33]. In the current study, all patients could tolerate and complete the ablation, and no serious complications occurred in any case. Only a few patients experienced side effects such as pain and local discomfort, which may be related to thyroid parenchymal edema and thyroid envelope heat injury after ablation [12]. In addition, the TSH level was slightly decreased in one patient, and the thyroglobulin antibody level was slightly increased in another patient 1 month after PLA. For both patients, thyroid function returned to normal at later follow-ups without any clinical treatment. We believe that these changes may have been due to thyroid tissue damage that was temporary and self-limited.

A recurrence rate of 2.86% (3/105 treated patients) was observed in our study. In one patient, a new PTMC lesion in the same lobe was detected, and metastatic central cervical lymph nodes were identified in two other patients after PLA. However, no case of distant metastasis was observed. Considering that the recurrence rates of PTMC were 1.4% at 5 years and 3.4% at 10 years after surgical treatment in the study by Ito et al. [34], our results indicate that the long-term efficacy of PLA for treating PTMC was not inferior to that of surgery. The cause of recurrence is likely related to the location of the lesion and the insufficient range of PLA and may also be related to the subtype of PTC. Studies with larger samples sizes are needed to clarify the causes of PTMC recurrence after PLA.

Our study has several limitations. First, although the number of samples in the present study is significantly greater than those in previous studies, there may still be bias in the results. Establishment of PLA as a treatment method for PTMC will require a prospective, multicenter large-sample study, in order to fully characterize the efficacy of PLA for treating PTMC [35]. Second, multifocality cannot be absolutely excluded without histological examinations. Third, ultrasound could not detect all metastases from PTMC after PLA because of the potential limitations of US in differentiating lymph nodes of central compartment. Finally, we did not obtain the final histopathology of these nodules, false-positive or false-negative results might occur when using FNAB before PLA. Treatment of PTMC by PLA is still in the trial stage, low-risk patients, such as those with small, single-onset lesions, no lymph node metastasis, and long-dormant lesions, should be selected for the exploration of thermal ablation of PTMC.

In conclusion, ultrasound-guided PLA was found to be a safe therapy for treating solitary PTMC lesions with a good therapeutic effect. Thus, this study provides valuable clinical evidence for the treatment of PTMC patients with PLA.

## Figures and Tables

**Table 1:** Clinical and treatment characteristics for 105 PTMC patients included in this study.

Characteristics (*n* = 105)	
Female : male, number (%)	74 (70.5) : 31 (29.5)
Age (years)	44.1 ± 12.2 (23–68)
Nodule location, *n* (%)	
Left lobe	44 (41.9%)
Right lobe	61 (58.1%)
Largest diameter of the nodule (mm)	6.34 ± 2.62 (4.5–9.8)
Nodule volume (mm^3^)	99.42 ± 84.01 (20.28–416.56)
Duration of ablation procedure (min)	2.78 ± 1.05 (2.2–4.8)
Gross energy (J)	505 ± 185 (396–864)
Follow-up (months)	65.4 ± 6.3 (60–96)

**Table 2:** Nodule diameter and volume before and after ultrasound-guided PLA treatment of PTMC as well as the volume reduction rate (VRR) at each follow-up timepoint.

Time	No. of nodules	Largest diameter (mm)	Mean volume (mm^3^)	VRR (%)	*P*
Pretreatment	105	6.34 ± 2.62 (4.5–9.8)	99.42 ± 84.01 (20.28–416.56)	—	—
1 hour	105	18.28 ± 4.37 (9.56–23.51)	1067.23 ± 868.76 (357.48–2087.38)	−781.14 ± 653.29 (-1386.62– -489.71)	<0.001
1 month	105	13.53 ± 3.79 (6.97–17.44)	761.15 ± 498.83 (165.34–1383.11)	−268.65 ± 179.57 (-399.73–-78.61)	<0.001
3 months	105	9.68 ± 2.63 (2.92–12.71)	376.42 ± 267.66 (68.28–653.19)	−98.39 ± 76.58 (-147.59–63.24)	<0.001
6 months	105	5.48 ± 2.46 (0–8.95)	137.48 ± 86.55 (0–273.37)	36.78 ± 30.32 (17.42–100.00)	0.002
12 months	105	1.95 ± 1.74 (0–4.28)	8.47 ± 7.91 (0–76.36)	75.55 ± 21.81 (59.41–100.00)	<0.001
18 months	105	0.23 ± 0.47 (0–0.91)	0.41 ± 1.62 (0–28.43)	96.79 ± 10.57 (83.36–100.00)	<0.001
24 months	103	0	0	100	<0.001
36–60+ months	103	0	0	100	<0.001

^∗^ *P* values relative to pretreatment volume.

## Data Availability

The data used to support the findings of this study are available from the corresponding author upon request.
